# Insights into evolutionary interaction patterns of the 'Phosphorylation Activation Segment' in kinase

**DOI:** 10.6026/97320630015666

**Published:** 2019-10-13

**Authors:** Adil Ahiri, Hocine Garmes, Crtomir Podlipnik, Aziz Aboulmouhajir

**Affiliations:** 1 Modeling and Molecular Spectroscopy Team, Faculty of Sciences, University Chouaib Doukkali, El-Jadida, Morroco; 2 Analytical Chemistry and Environmental Sciences Team, Department of chemistry, Faculty of Science, University Chouaib Doukkali, El Jadida, Morroco; 3 Faculty of Chemistry and Chemical Technology, University of Ljubljana, Ljubljana, Slovenia; 4 Extraction, Spectroscopy and Valorization Team, Organic synthesis, Extraction and Valorization Laboratory, Faculty of Sciences of Ain Chock, Hassan II University, Casablanca, Morocco

**Keywords:** Kinase, activation segment, phosphorylation, structural variation, interaction variation

## Abstract

We are interested in studying the phosphorylation of the kinase activation loop, distinguishing the passage from the unphosphorylated to the phosphorylated form without allostery. We performed an interaction study to trace the change of interactions between the activation segment and the kinase catalytic core, before and after phosphorylation. Results show that the structural changes are mainly due to the attraction between the phosphate group and guanidine groups of the arginine side chains of RD-pocket, which are constituted mainly of guanidine groups of the catalytic loop, the β9, and the αC helix. This attraction causes propagation of structural variation of the activation segment, principally towards the N-terminal. The structural variations are not made on all the amino acids of the activation segment; they are conditioned by the existence of two beta sheets stabilizing the loop during phosphorylation. The first,β6-β9 sheet is usually present in most of the kinases; the second, β10-β11 is formed due to the interaction between the main chain amino acids of the activation loop and the αEF/αF loop.

## Background

**Figure 1:**
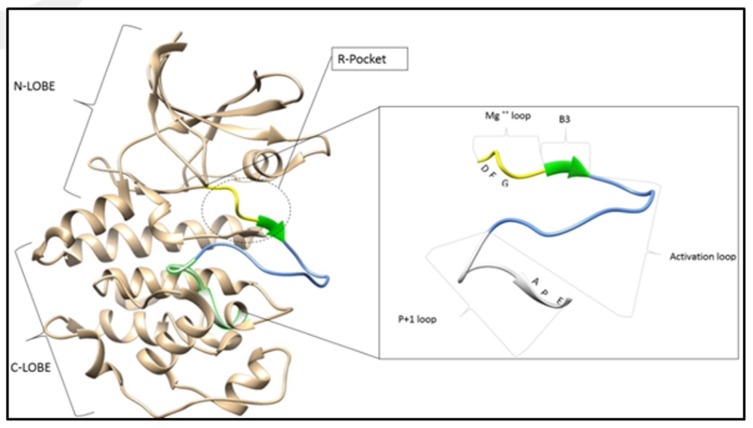
The P-kinase domain of GSK3B showing the activation segment and its various constituents

The reversible interplay between protein kinases and Phosphatases has an important role in cellular signalization [1]. Protein kinases are enzymes which mediate the transfer of γ-phosphate group (PO32-) from ATP to target protein substrates [1]. On the contrary, phosphatases remove phosphate groups from the phosphorylated protein substrate [1]. The protein kinases have short consensus sequences in common, which define the catalytic subunit of about 270 amino acids, distributed in two lobes: the N terminal lobe (about 80 residues) consisting essentially of a β-sheet with five strands and a Cα helix [2]. The loop connecting the first two strands β is P-loop. The C-terminal lobe (about 190 residues) consists mostly of α-helices and two essential close loops, which are catalytic loop and activation loop [2]. The latter belongs to the activation segment which runs from the conserved DFG (Asp-Phe-Gly) in the magnesium binding loop to the conserved APE (Ala-Pro-Glu) and includes β9, the activation loop and the P+1 loop, moving from N-terminal to C-terminal anchor points [3]. The conformation of the whole activation segment controls kinase activity [3]. The two lobes are connected by a so-called hinge region ensuring their mutual flexibility [2].The phosphorylation of the substrate occurs in the catalytic site or active site, which is at the intersection of N and C lobes (Figure 1). This site contains two juxtaposed pockets to receive the ATP and the substrate, respectively [2]. The regulation by phosphorylation of the kinase is essential for its catalytic competence [3]. It can take place either directly by modulating the ATP binding, or the substrate binding on their catalytic site indirectly, by the displacement, to blocking key elements of the catalytic, or regulation domain [3]. The kinase can also be catalytically inactive due to the displacement of key elements participating in the catalytic domain, which is then allosteric regulation [3]. The activation of some kinases requires phosphorylation of their activation segment, particularly their activation loop which is a real phosphorylation regulatory site [3]. The activation loop is involved in the kinase regulations which gives an essential role in the adoption of an active conformation. SLK phosphorylation at T183 and S189 levels is known [4]. Phosphorylation plays an important role in the activation and signalling processes of SLK [4]. Besides, the regulatory effect extends to the catalytic environment by the activation segment. Researchers have recorded a deregulation of MAPK at low pH due to a structural rearrangement of the activation segment [5]. The latter may also have an auto-inhibitory action because of substrate blocking and stabilisation of an inactive conformation of the αC helix, such as the case of NDR1 in its non-phosphorylated state [6]. What is more, one of the most important mechanisms related to the activation segment is auto phosphorylation, where kinases dimerize and cause phosphorylation of the activation segment [7,[8,9]. So, the exchange of activation segment has proven to be a very important action in the auto phosphorylation mechanism [11], which can occur in one or both directions [12]. It is remarkable that autophosphorylation does not recognize the consensus sequences of substrates, which gives us a unique phosphorylation mechanism [10]. Concerning the physical aspect, the activation segment starts from the conserved motif DFG, which is the part of the activation loop, to the conserved motif APE (Figure 1) [3].The N-terminal and C-terminal anchor points of the activation segment take place despite the folding of the activation loop under the effect of the phosphorylation of one of the kinase residues [3]. In fact, in many kinases, the interaction of the phosphorylated residue with the activation loop gives two states. One concerns the aspartate of the DFG motif pointing in the ATP binding site and coordinates two Mg2+ ions (active state) DFGin, and the other concerns DFG pointing out of the ATP binding site (inactive state, DFGout) [13]. When the N-terminal or the C-terminal anchor points are disturbed, the kinase is in an inactive state. In general, it is commonly accepted that in the cell environment, kinases pass between the catalytically active conformation and inactive conformation [13]. There are also other secondary phosphorylation sites, both upstream and downstream of the primary site, improving the regulation of kinase activity [3]. Protein kinases are subdivided into subfamilies according to their different catalytic domains specificity and to target amino acids, knowing that tyrosine phosphorylation has attracted more interest in biomedical research thanks to its relation to human disease via the dysregulation of receptor tyrosine kinases (RTKs) [14]. Indeed, currently, 37 kinase inhibitors have received FDA approval for the treatment of cancer, and about 150 kinase-targeted drugs are in various clinical phase trials [14]. The human protein kinases numbering 518, constitute one of the largest family of the human genome, representing 1.7% of the genome [15].These protein kinases are subdivided into two categories: the first, with 478 families, has typical eukaryotic catalytic domains (ePKs), and the second, contains 40 families with atypical catalytic domains [16]. The study of sequence identities of the catalytic domains of ePKs revealed 491 catalytic domains divided into nine groups [17]. Frequently, the non-catalytic domains are misnamed as regulatory domains, although the regulation and phosphorylation can also take place in the catalytic domain in this case [3]. Several structural studies have reported the interaction between the phosphorylated amino acid and the catalytic body without integrating the whole activation segment [3]. We report here the correlation of the "phosphorylation activation segment" P.A.S. and more specifically activation loop, with principal residues in kinase activity. We further explain the structural changes due to the P.A.S-kinase catalytic core interaction, benefiting from increasingly abundant databases of protein structures, and avoiding expensive quantum or dynamic calculations. Hence, a differential structural analysis between phosphorylated and non-phosphorylated P-kinases (kinase catalytic core) is completed.

## Methodology

### Dataset preparation:

The P-kinases sequences were extracted from the UNIPROT database [18], and their 3D structures were identified in the PDB RCSB database [19] by BLASTP. During alignment with BLAST, we have chosen an E-value=0 to avoid all the alignments randomly and to be able to extract structures principally with identical sequences from the UNIPROT database [20]. The PhosphoSite database [21] allows us to locate the phosphorylated amino acids in kinases sequences. We have retained only structures with resolved Activation segments, and their visualization was made in the Chimera program [22]. For a successful comparison between phosphorylated and non-phosphorylated kinases, it was necessary to build a homogeneous database, thereby removing different factors that may interfere with phosphorylation. Therefore, a selectivity protocol integrating three filters was set up. The first filter consists of comparing P-kinases of the same stoichiometry [23]. While the second filter annihilates the allosteric effect in the active sites, by eliminating all structures having ligands positioned in their allosteric sites [24]. We selected all the ligands possessing the best value of interaction fingerprint compared with ATP for further studies [25]. The third and last filter, called the RMSD (Root-Mean-Square Deviation) Cα clustering effect, which measures the main chain carbons fluctuations, has the role of avoiding, among the structures proposed in the PDB for the same kinase, all of the very fluctuating structures giving rise to a Cα RMSD exceeding 2 Åduring their comparison [26].

### Dataset treatment:

After the selection of the phosphorylated and not phosphorylated structures by the different filters, we focused only on the catalytic domain, using the annotation provided by PROSITE [27]. Then, the missing sequences of this domain, which are not solved by XRAY, are modelled by MODELLER on the basis on PDB of the same class previously collected during preparation [28] and validated (checked) by Meta server SAVES. The next step consists of rectifying mutations using the most crucial score of Dynamics Rotamer Library [29].

### Dataset analysis:

We have studied the conservation of secondary structures in the activation segment for selected P-kinases, before discussing the difference between compared 3D structures of the unphosphorylated structure considered as a reference, and phosphorylated structure for the extraction of the phosphorylation effect. For this, alignment of sequences, and a superposition of their secondary structures have been carried out.

We have performed an analysis of the cartesian deviations of the backbone chain by RMSDbb, side chain, by RMSDsc, and also the deviations of the dihedral angles σ (ϕ), σ (ψ) of each amino acid of our kinase to detect structural disparities between the phosphorylated and non-phosphorylated activation loop structures [30]. We have retained only variations which exceed 2 Å for RMSDbb and RMSDsc [31] and 20 ° for the dihedral angles [32]. Finally, to identify the impact of phosphorylation of the activation loop on the modes of its interaction with the remaining P-kinase motifs, we have attempted to enumerate the amino acids of activation loop which are essential for such interactions, using the PICI script [33].

## Results and discussion

The structures retained by the filters used during the study of the structural differentiation between P-kinase, before and after phosphorylation of the activation loop, are listed in Table 1. The interaction tables can be found in the supplementary material (see PDF version at pages 673-676). Cyclin-dependent kinase 2 (CDK2) [34] is an essential component of the cell cycle machinery, with maximal activity during S phase. As its name indicates, its functionality depends on the presence of cyclin [35]. Phosphorylation at T14 or Y15 residues of CDK2 causes inactivation of CDK2, whereas phosphorylation at T160 increases its activity [36-37]. The importance of the phosphorylation of the latter was confirmed during its mutation to alanine, leading to a decrease of the CDK2 activity five times [38].The phosphorylation of CDK2 at T160 leads this amino acid to interact by hydrogen bonds with R50 (αC helix), R126 (catalytic loop), R150 (β9) and Y180 (loop αEF/αF). T160 is exhibiting deviations of the backbone chain (RMSFbb) and side chain (RMSFsc) exceeding 3Å and a deviation of the dihedral angle σ(ψ) of about 80°; the same interactions were obtained by L. N. Johnson [39]. We can prove the flexibility of the activation loop amino-acids compared to all explored amino acids (from F4 to F286), due to their ability to move easily to other amino acids according to specific interactions. The deviation will rise to the disappearance of interactions between the amino acids of activation loop (V156, R157, T158) and those (G176, C177, K178, Y180) of the loop αEF/αF, which will leads to the total demolition of the secondary structure β10-β11.

Glycogen synthase kinase three beta(GSK3B) is a protein of the GSK family of CMGC group [40], phosphorylates glycogen synthase to inactivate, participate in the Wnt signaling pathway [41], which is involved in energy metabolism and neuronal cell development [41]. Phosphorylation of GSK3 at Y216 position acts as an activator of the protein [42]. Scientists believe that the phosphorylation of Y216 facilitates substrate phosphorylation, but it is not strictly necessary [42]. For the phosphorylation of the activation loop at the Y216 of the structure GSK3β kinase causes an attraction with the guanidine groups of the two arginines 220, 223 of the P+1 loop. The attraction of the two groups mainly generated by a deviation of the side chain of 4.3 Å and the dihedral angle of σ (ϕ): 27.5° and σ(ψ): 17.1°, which agrees with the published results of Krupa, who talks about the secondary phosphorylation site [43]. GSK3β kinase catalytic domain mainly shows modifications of the dihedral angles σ (ϕ) and σ(ψ) at the level of the amino acids: R209 to Y216 of the activation loop. Following these structural modifications, the propagation of the phosphorylation affects only a few amino acids of the activation loop, from the phosphorylated residue to the N-terminal R209, and does not affect any amino acid of the C-terminal of the activation segment after phosphorylation. We also note that there is an insufficient variation in interactions, whereas there is a conservation of secondary structures (β6-β9) and (β10-β11). Kinase reactive subdomains show no significant interaction variation. 

Mitogen-activated protein kinases (MAPK) belong to the group of CMGC [44], responding to stress stimuli such as ultraviolet irradiation, cytokines, heat, and osmotic shock and participate in cell differentiation, apoptosis, and autophagy [45]. Activation of this enzyme occurs by dual phosphorylation of T180 and Y182 [46]. It is 10-20 times more active than MAPK14 which is phosphorylated only on T180 [46], whereas MAPK14 phosphorylated on T182 alone is inactive [46]. The dual phosphorylation of T180 and Y182 of MAPK14 causes two unique attractions, one for T180 to the guanidine groups, R149 of the catalytic loop, and N-terminal residues R67 and R70 of the Cα helix [47]. In MAPK14 kinase, there is an appearance of the hydrogen bonds between amino acids of Mg2+ loop (A172, R173, H174, T175) and the amino acids of subdomain VIB that contains the catalytic loop (D145, I146, I147, H148, R149). The dual phosphorylation causes a broad propagation along the activation loop, but also some amino acids Mg2+ loop (A172, R173, and H174) since the two β sheets (β6-β9 and β10- β11) will not support the activation loop of the unphosphorylated structure. This propagation gives rise to a release of the active site, following dilation of the activation loop. The kinase emphasizes substantial cartesian modifications at the level of the activation segment, ranging from 3.1 to 12.1 Å, which caused an increase in the number of interactions, that is rational with its importance in the stability of the structure.

3-Phosphoinositide-dependent kinase 1 (PDK1) is a kinase of the AGC group of the PKB family which contains a PH domain [48]. Since the pleckstrin homology (PH) domain promotes binding PDK1 to the plasma membrane [49]. It is involved in a large variety of processes, including cell proliferation, differentiation, and apoptosis [50]. Auto phosphorylation of the activation segment at S241 is necessary for PDK1 activity [50]. Phosphorylation of PDK1 at S241 deviates its RMSD in the order of 3.6 Å at the main chain and 4.8 Å in the side chain, and of the order of 144.8° at the dihedral angle σ (ψ). This deviation induces an attraction between the phosphate group with the guanidine groups of the two R129 arginines of the Cα and R204 of the catalytic loop. This last result coincides with the results published by Komander, but which adds interaction with K128 of β9 and T126 of Cα helix [51]. The phosphorylation of PDK1 kinase does not give rise to a significant structural change, following conservation of secondary structures, but it gives rise to a change of interactions between Q236, A237, R238 of the activation loop and C260, S258, A259 of the loop αEF/αF. This phosphorylation is followed by some changes in the interactions of several amino acids between the activation loop and the catalytic body of the kinase, marking the disappearance of hydrogen interaction of R204 of the HRD motif, with A239 and a change at R238 by hydrogen interactions with S258 and A259. Additionally, there is an appearance of hydrogen bonds at the amino acid level F242 with R204.

Spleen tyrosine kinase (Syk) is a cytoplasmic tyrosine kinase SYK family [52], containing two SH2 domains [53]. It plays a central role in the response of the B-receptor (BCR) [54]. The activation of this kinase requires the detachment of the two SH2 domains and many phosphorylations at this level [55]. in addition to double phosphorylation at positions Y525 and Y526 in the activation segment [56]. Moreover, SYK kinase dual phosphorylation does not lead to a significant change in interactions and does not affect specific sub-domains, except in the case of the disappearance of two D512 and G514 hydrogen interactions of the motif DFG with N381 and F382. This last amino acid is marked by the most significant deviation at the dihedral angle σ (ψ) = 167.4°. The low number of interactions may be due to the stability of the activation loop in the two cases of phosphorylation, since the secondary structure of the unphosphorylated form has the two sheets (β6-β9 and β10-β11). Single phosphorylation does not result in a large propagation of this effect all along the activation loop, because this propagation only affects the C-terminal two amino acids in the case of single phosphorylation (T530, H531). Phosphorylation of PAK1 kinase does not result in a fluctuation transfer at the activation loop, which results in a small number of interactions that do not affect specific subdomains. The activation loop is stabilized with the beginning of the formation of the β10-β11 sheet, and the β6-β9 sheet.

Aurora is a member of the AUR kinase family [57]. The kinase is located on mitotic centrosomes and microtubules, required for centrosome maturation [58]. Activation of Aurora requires binding of the TPX2 complex [59], but is enhanced by phosphorylation at position T287 and is suppressed when position T288 is also phosphorylated [60]. In case of AURORA kinase monophosphorylation at T287, the activation loop remains stabilized with the beginning of the formation of the β10-β11 sheet and the β6-β9 sheet. This stability is reflected in the small fluctuation of the entire segment. All these fluctuations do not prevent some structural changes scattered between the two lobes and marked by the conservation of multiple interactions between Q177 and W277, and the disappearance of a hydrogen interaction with G276, which belongs to the DFG motif. As regards monophosphorylation at T288, in an earlier work by Bayliss, we have found a difference in the analyzed structure,, since it is complexed with TPX2. We note that we do not have the same interactions with T288, whereas there is the appearance of interactions that resemble the case of T287 phosphorylation. This finding allows us to say in the case of the work of Bayliss, that these interactions are not due to the phosphorylation but to the complexation of TPX2 [1]. For the doubly phosphorylated structure, it is noted that R285, of the phosphorylated structure T288, binds with R180 and R255, the same goes with those where the phosphorylated structure binds to T287 as if there exists some competition between the T287and T288 phosphorylated structures.

## Conclusion

Results show that the structural adaptation of the activation loop after phosphorylation is mainly due to hydrogen bonds formed between the phosphate group with amino groups (R-NH2) of lysines or with guanidine groups (R-CH5N3) of arginines. The multiplicity of these phosphate groups interactions represent anchors, which stabilize the activation loop. The strongest anchor belonging to the N-terminal concerns arginines or lysines of the Cα and β9 and also arginines of the HRD motif, as well. The second remarkable anchor, which belongs to C terminal, concerns arginines or lysines of the αEF and leads to a rearrangement of the loop P+1 amino acids. Moreover, these anchors allow interactions propagation towards the N-terminal lobe of the activation segment. Further, it should be noticed that the activation loop stability is conditioned by the existence of interactions between the main chains of the activation loop and the αEF/αF loop. Besides, the structures containing β6-β9 sheets in the N-terminal or β10-β11 in the middle of the activation loop strengthen its stability during phosphorylation. Finally, we find out that interactions' variations are acting on the most essential regions at the level of kinases with the hydrogen bonds, affecting the most conserved motifs in kinases: DFG for the SYK kinase, APE for the PAK1 kinase and HRD for CDK2, MAPK14, PDK1, PAK1, AurA as well as other ones which react on the important loops, like GSK3β on Mg2+ loop and MAPK14 on P+1 loop.

## Figures and Tables

**Table 1:** Dataset of phosphorylated and unphosphorylated structures compared

GROUPE	KINASE (FAMILY)	Stoichiometry	ID	Species	Ligands	MUTATION
CMGC	CDK2 (CDK)	AB	4EOQ_A	Homo sapiens	ATP, MG, TPO_160_	
		AB	1FIN_A	Homo sapiens	ATP	
	GSK3B (GSK)	A1	2O5K_A	Homo sapiens	HBM	
		A1	2OW3_A	Homo sapiens	BIM, PTR_216_	Y216X
	MAPK14/P38 (MAPK)	A1	1P38_A	Mus musculus		
		A1	3PY3_A	Mus musculus	PTR_182_,TPO_180_	
AGC	PDK1 (PKB)	A1	2BIY_A	Homo sapiens	ATP, GOL, SO4	S241A
		A1	3RCJ_A	Homo sapiens	3RC, SEP_241_	Y288G, Q292A
TK	Syk (Syk)	A1	4YJT_A	Homo sapiens	4DQ, GOL, PTR _525_, _526_	
		A1	5CXH_A	Homo sapiens	55M	
		A1	3SRV_A	Homo sapiens	GOL, PTR_525_,S19	
		A1	4XG3_A	Homo sapiens	X3G	
STE	PAK1 (STE20)	A1	3FXZ_A	Homo sapiens	FLL, TPO_423_	K299R
		A1	1YHW_A	Homo sapiens		K299R
OTHER	AurA (AUR)	A1	1OL7_A	Homo sapiens	ADP, MG, TPO_287_,_288_	
		A1	5DNR_A	Homo sapiens	ATP, MG, SO4, TPO_288_	
		A1	5DT3_A	Homo sapiens	ATP, MG, SO4, TPO_287_	
		A1	4DEE_A	Homo sapiens	ADP, MG	T287D
